# Hyperglycemia Induces Oxidative Stress and Impairs Axonal Transport Rates in Mice

**DOI:** 10.1371/journal.pone.0013463

**Published:** 2010-10-18

**Authors:** Ruchi Sharma, Eric Buras, Tomoya Terashima, Faridis Serrano, Cynthia A. Massaad, Lingyun Hu, Brittany Bitner, Taeko Inoue, Lawrence Chan, Robia G. Pautler

**Affiliations:** 1 Translational Biology and Molecular Medicine, Baylor College of Medicine, Houston, Texas, United States of America; 2 Department of Molecular Physiology and Biophysics, Baylor College of Medicine, Houston, Texas, United States of America; 3 Department of Medicine, Baylor College of Medicine, Houston, Texas, United States of America; Cornell University, United States of America

## Abstract

**Background:**

While hyperglycemia-induced oxidative stress damages peripheral neurons, technical limitations have, in part, prevented *in vivo* studies to determine the effect of hyperglycemia on the neurons in the central nervous system (CNS). While olfactory dysfunction is indicated in diabetes, the effect of hyperglycemia on olfactory receptor neurons (ORNs) remains unknown. In this study, we utilized manganese enhanced MRI (MEMRI) to assess the impact of hyperglycemia on axonal transport rates in ORNs. We hypothesize that (i) hyperglycemia induces oxidative stress and is associated with reduced axonal transport rates in the ORNs and (ii) hyperglycemia-induced oxidative stress activates the p38 MAPK pathway in association with phosphorylation of tau protein leading to the axonal transport deficits.

**Research Design and Methods:**

T_1_-weighted MEMRI imaging was used to determine axonal transport rates post-streptozotocin injection in wildtype (WT) and superoxide dismutase 2 (SOD2) overexpressing C57Bl/6 mice. SOD2 overexpression reduces mitochondrial superoxide load. Dihydroethidium staining was used to quantify the reactive oxygen species (ROS), specifically, superoxide (SO). Protein and gene expression levels were determined using western blotting and Q-PCR analysis, respectively.

**Results:**

STZ-treated WT mice exhibited significantly reduced axonal transport rates and significantly higher levels of ROS, phosphorylated p38 MAPK and tau protein as compared to the WT vehicle treated controls and STZ-treated SOD2 mice. The gene expression levels of p38 MAPK and tau remained unchanged.

**Conclusion:**

Increased oxidative stress in STZ-treated WT hyperglycemic mice activates the p38 MAPK pathway in association with phosphorylation of tau and attenuates axonal transport rates in the olfactory system. In STZ-treated SOD-overexpressing hyperglycemic mice in which superoxide levels are reduced, these deficits are reversed.

## Introduction

Diabetes affects more than 300 million people worldwide [1]. While the effect of hyperglycemia has been well characterized in peripheral neurons, we know little about the effect of hyperglycemia on the central nervous system (CNS) partly due to the limitation of noninvasive dynamic imaging techniques to assess neuronal function [2]. Even though olfactory dysfunction is indicated during diabetes, little is known about the effect of hyperglycemia on the olfactory system. [3]–[5]. We previously described measurement of axonal transport rates in the olfactory bulbs in a mouse model of Alzheimer's disease [6], [7]. In this study, we utilized manganese enhanced MRI (MEMRI) to determine the effect of short-term hyperglycemia-induced oxidative stress on axonal transport rates in the olfactory receptor neurons (ORNs) in mice.

Oxidative stress is regulated by the levels of reactive oxygen species (ROS), which includes superoxides, hydroxyl radical, and hydrogen peroxide. Normally, ROS produced during cellular respiration are removed by mitochondrial antioxidant enzymes such as superoxide dismutase 2 (SOD2), which converts superoxide to hydrogen peroxide, which in turn is converted to water and oxygen by glutathione, and catalase. During hyperglycemia, excess glucose levels induce over-production of ROS in the mitochondria, which subsequently causes depletion of SOD2 and leads to cellular injury [2], [8]–[10].

Whereas hyperglycemia-induced oxidative stress damages peripheral neurons, overexpression of SOD2 reduces the superoxide load and prevents hyperglycemia-induced cellular injury in dorsal root ganglia (DRG) cultures [10]. Additionally, overexpression of SOD2 reduces symptoms of neuropathy in streptozotocin (STZ) treated C57Bl/6 mice [10]. Furthermore, the mechanism of neuronal injury due to hyperglycemia-induced oxidative stress has been shown to occur through the activation of p38 mitogen-activated protein kinases (MAPK) [9]. Activation of the p38 MAPK signaling cascade is associated with cellular response to cytokines, irradiation, and oxidants [11]–[13]. In peripheral neurons, hyperglycemia-induced activation of p38 MAPK pathway is associated with reduced motor nerve conduction velocity [11] and apoptosis [14].

We hypothesize that (i) hyperglycemia induces oxidative stress and is associated with reduced axonal transport rates in the CNS. We also hypothesize that hyperglycemia-induced oxidative stress activates the p38 MAPK pathway in association with phosphorylation of tau protein leading to axonal transport deficits in CNS neurons. Last, we hypothesize that reduction of oxidative stress through the overexpression of SOD2 should ameliorate these effects.

To address these hypotheses, we utilized a dynamic, *in vivo*, MRI methodology, MEMRI, to measure axonal transport rates non-invasively [6], [7]. Manganese ion (Mn^2+^) is a paramagnetic MRI contrast agent that instills positive contrast in spin-lattice (T_1_) weighted MRI images [15]. Additionally, Mn^2+^ is known to enter the cells via voltage-gated calcium channels [16]. Once in the cells, Mn^2+^ is packaged into vesicles and transported along microtubules via fast axonal transport and released at the synapse [6]–[7], [17]–[22]. The rates of Mn^2+^ transported along the axons are reflective of the fast axonal transport rates [6]–[7], [17]–[22]. Dynamic T_1_-weighted MEMRI enables trans-synaptic, *in vivo*, MRI detectable neuronal tract tracing to map neuronal pathways [6]–[7], [17]–[22].

In this study, we utilized two different animal models: (i) STZ-induced hyperglycemia mice and (ii) a heterozygotic SOD2 overexpressing STZ-treated hyperglycemia mice. Overexpression of SOD2 reduces superoxide load in DRG neurons [10] and in hippocampal neurons [23] and prevents cellular injury. The SOD2 overexpressing mice are a transgenic line that overexpress human SOD2 gene driven by the β-actin promoter [24]. We first determined axonal transport rates in STZ-treated wildtype (WT) and SOD2 overexpressing mice. We next determined the levels of ROS in the different groups of mice. Last, we determined the protein and gene expression levels of p38 MAPK and tau. Our findings suggest that hyperglycemia-induced oxidative stress instills axonal transport deficits in ORNs through the phosphorylation of p38 MAPK and tau.

## Methods

### Animal Models, MRI Imaging and Processing

Two to four month-old WT and SOD2 overexpressing mice on a C57Bl/6 background were utilized for this study. *STZ treatment*: Hyperglycemia was induced by an intraperitoneal (i.p.) injection of 170 µg/g of STZ. Control animals received vehicle injections of 0.01 M sodium citrate. Mice with fasting blood glucose levels >200 mg/dl or non-fasting blood sugar >250 mg/dl (on the third day post-STZ treatment), were considered hyperglycemic. Experiments were performed 1-week post-injections. *Insulin treatment*: In order to first validate the effect of STZ-induced hyperglycemia and to rule out possible effects of STZ-toxicity on the axonal transport rates we treated the hyperglycemic mice with insulin. Linbit insulin implants were utilized for the insulin treatment. (LinShin, Inc, Canada). After confirmation of high glucose in STZ treated animals used, half of a Linbit tablet was implanted subcutaneously on the back of the mice. The feeding glucose levels were checked twice during the following week. If the glucose levels were over 250 mg/dl, an additional half tablet of insulin was implanted. *Glucose treatment*: To validate that the changes in axonal transport rates were from increased glucose levels, chronic hyperglycemia was induced by injecting 25% D-glucose (i.p.) 4 times a day for 5 days and a single injection on the sixth day before imaging at a dose of 1.25 g/kg mouse. All mice were housed at Baylor College of Medicine's transgenic mouse facility in compliance with the National Institutes of Health (NIH) guidelines for Care and Use of Laboratory Animals. The mouse facility is kept on a 12-hour light–dark cycle, with a regular feeding and cage-cleaning schedule. All experiments were approved and conducted in compliance with BCM's Institutional Animal Care and Use (IACUC) regulations under animal protocol #AN-3334.

Mn^2+^ administration was conducted as we have previously performed [6]–[7], [17]. Briefly, animals were anesthetized with 5% isoflurane. Anesthetized mice were nasally lavaged with 4 µl of 0.75 mg/ml MnCl_2_ dissolved in nanopure water. The olfactory system is easily accessible and minimally invasive. Following Mn^2+^ administration, the animals were allowed to recover. One hour post-lavage, animals were re-anesthetized with 5% isoflurane and imaged. The body temperature was maintained at 37° utilizing a heated air system (SA Instruments, Inc, Stony Brook, NY) and maintained under anesthesia with 2% isoflurane in 100% O_2_. The respiration was monitored with a pressure pad placed under the animal and temperature was monitored by a rectal probe (SA Instruments, Inc, Stony Brook, NY). Respiratory rates and temperature were monitored using the Model 1025 Small Animal Monitoring and Gating System software (SA Instruments, Inc, Stony Brook, NY).

**Figure 1:**
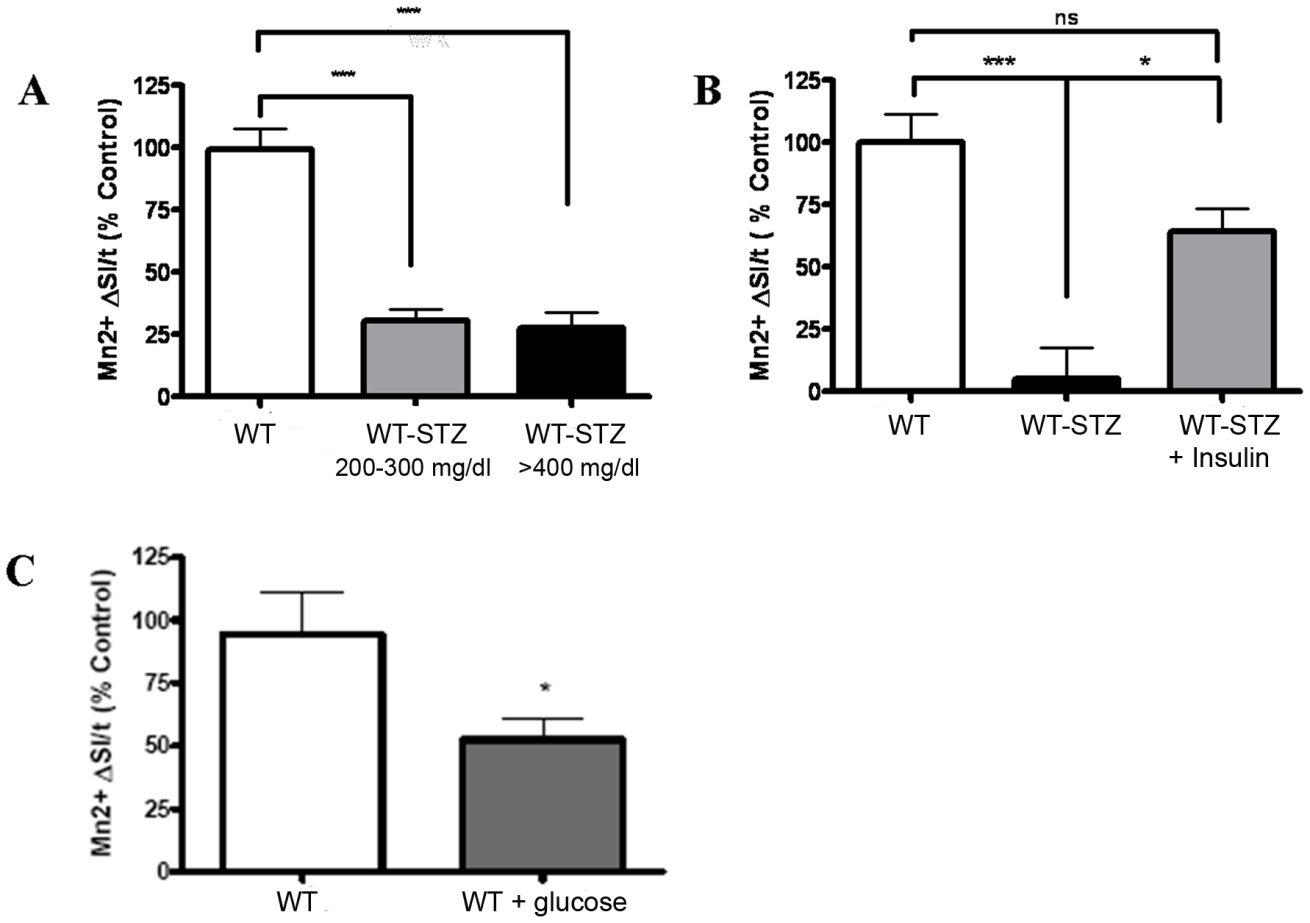
STZ-induced hyperglycemia impairs axonal transport in the olfactory receptor neurons. (A) Graph depicts axonal transport rates (depicted as Mn^2+^ ΔSI/t on Y axis, where “SI” is signal intensity and “t” is time) of Mn^2+^at 1 week post-STZ treatment for WT(n = 3) and WT-STZ with fasting glucose levels 200–399 mg/dl (n = 3), and >400 mg/dl (n = 5). (B) Graph depicts the changes in axonal transport rates for WT (n = 4), WT-STZ (n = 3), and WT-STZ+insulin treated mice (n = 4). (C) The graph represents changes in axonal transport rates in a mouse model of WT-STZ (n = 5) as compared to WT mice (n = 4). Statistical analysis: One way ANOVA, Tukey's post test for more than 2 groups and Students t test to compare 2 groups. * p<0.05, ** p<0.01, *** p<0.001/WT  =  wildtype control. WT-STZ  =  Wildtype treated with streptozotocin.

A series of T_1_-weighted images were acquired utilizing a 9.4T, Bruker Avance Biospec Spectrometer, 21 cm bore horizontal scanner with a 35 mm volume resonator (Bruker BioSpin, Billerica, MA). The imaging parameters to acquire spin-echo 2D images were as follows: repetition time, (TR)  = 400 ms; echo time (TE)  = 10.2 ms; FOV = 3.0 cm; slice thickness  =  1 mm; matrix dimensions  = 128×128; number of averages, NA = 2; and number of cycles  = 15; each cycle took approximately 2 min 8 sec to acquire using Paravision software (Bruker BioSpin, Billerica, MA). Core temperature was maintained at 37°C during scanning. Regions of interest (ROI) were localized on axial slice. The axial slice location was similar between all the mice and was consistently located 1 mm in front of the posterior of the olfactory bulb (OB). For the initial axonal transport studies (Figure 1A), wildtype (WT), N = 3; WT treated with STZ (WT-STZ) with glucose levels  = 200–299, N = 3; for WT-STZ with glucose levels  = >300, N = 5. For the axonal transport studies in which insulin was administered (Figure 1B), WT, N = 3; WT-STZ, N = 3; WT-STZ treated with an insulin pellet. N = 4. For the chronic hyperglycemia studies (Figure 1C), WT, N = 4; WT + glucose, N = 5. For the SOD-2 experiments, WT, N = 12; WT-STZ, N = 4; SOD-2 overexpressing animals (SOD-2), N = 4; SOD-2 treated with STZ (SOD2-STZ), N = 4.

Signal intensities at the ROI were normalized to un-enhanced muscle from the same slice. The ROI was identified as we have previously performed [6], [7]. Briefly, the ROI was identified by locating the midpoint of the length of the OB and then extending a perpendicular line from the midpoint out to the olfactory neuronal layer. The normalized signal intensity was plotted versus time. The slope of the best fit linear regression curve indicated the rate of axonally transported Mn^2+^.

### Histology and Biochemistry

Dihydroethidium (DHE) was obtained from Invitrogen (Carlsbad, CA). Animals were treated with a single i.p. injection of 27 mg/kg of DHE. At 24 hours, DHE-injected animals were cardiac-perfused with 4% paraformaldehyde, the mice heads were decalcified with 0.24 M ethylenediaminetetraacetic acid for 8–10 days. The heads were then sectioned on a cryostat and mounted with Vectashield H1200 containing DAPI (Vector Laboratories, Burlingame, CA). Sections were evaluated for ethidium fluorescence (Excitation- 488 nm, Emission 590 nm, exposure time 100 ms) and DAPI (Excitation 405 nm, Emission 420–480 nm, exposure time 20 ms) using a Zeiss fluorescence microscope. Ethidium fluorescence (indicative of superoxide levels) was quantified in the nasal epithelium with the NIH software, ImageJ (Bethesda, Madison). We determined the ethidium fluorescence intensity using a 50×50 pixels square ROI identified on 1300×1030 pixels, RGB, 40× magnification images of nasal cavity sections and normalized the DHE mean signal intensity to the corresponding DAPI intensity. Five ROIs per section and 1–4 sections per animal were analyzed. Sections were randomly selected from the area spanning the depth of the nasal cavity. WT, N = 4, WT-STZ, N = 6; SOD2, N = 4; SOD-STZ, N = 4.

The OBs from mice brains were dissected fresh on ice and homogenized in lysis buffer (137 mM NaCl, 20 mM Tris-Base pH 7.6, 5 mM EDTA, 50 mM NaF, 2 mM NaVO_4_, 1 mM PMSF, and protease and phosphatase inhibitor cocktails from Sigma–Aldrich) using a sonic dismembrator (Fisher Scientific, Waltham, MA). The homogenates were then centrifuged at 10,000 g for 5 minutes and supernatant was used to assay for phosphorylated and total p38 MAPK and tau proteins. Total protein from the supernatant was measured using a Bradford assay (Bio-Rad, Hercules, CA). Proteins were equally loaded in each well of 10% polyacrylamide gel and separated by SDS/PAGE and then transferred onto a nitrocellulose membrane (Bio-Rad, Hercules, CA). The membranes were blocked with 5% milk and probed with 1∶1000 dilution, anti-phospho-p38 MAPK, anti-p38 MAPK (Cell Signaling, Danvers, MA), anti-phospho-Tau (threonine 205, serine 202) (MILLIPORE, Bellerica, MA), 1∶10,000 dilution, anti-Tau (DAKO, Denmark), 1∶30,000 dilution anti-α-tubulin (Sigma), and 1∶10,000 anti-β-actin antibodies (Sigma-Aldrich, St. Louis, MO). Membranes were next incubated with near-infrared fluorescent secondary antibodies from LI-COR (Lincoln, NE) (anti-rabbit for phospho and total p38 MAPK and Tau proteins and anti-mouse for α-tubulin and β-actin). Bands were visualized using LI-COR Odyssey machine. Anisomycin treated C6 cell extracts (Cell Signaling, Danvers, MA) were used as a positive control for phosphorylated p38 MAPK protein analysis. Brain homogenate from tau knockout mouse was used as a negative control for phosphorylated Tau protein assays. Given the multiple bands appearing on the p38 immunoblots, the specific band corresponding to phospho-p38 was identified by comparison to the positive control. Phospho-p38 yielded a double band at around 38KD, both of which were analyzed for densitometry and reported as a bar graph. For the western blot analysis of p38, WT, N = 7; WT-STZ, N = 5; SOD2, N = 5; SOD2-STZ, N = 5. For the total p38 protein levels, WT, N = 7; WT-STZ, N = 7; SOD2, N = 6; SOD2-STZ, N = 5. For the western blot analysis of tau, WT, N = 7; WT-STZ, N = 5; SOD2, N = 5; SOD2-STZ, N = 5.

Frozen brain samples were placed into TRI Reagent (Ambion, Austin, TX) and quickly homogenized in a dounce tissue homogenizer. RNA was isolated using the RiboPure kit (Ambion) per manufacturer's instructions. RNA quality was verified by observing bands corresponding to 28S, 18S and 5S ribosomal RNA on denaturing agarose gel and ensuring an approximate 2∶1 intensity ratio between 28S and 18S bands. RNA concentration was determined via absorption of ultraviolet light at 260 nm in a DU530 spectrophotometer (Beckman, Brea, CA). Total RNA mass was normalized across samples for the cDNA synthesis reaction. cDNA was synthesized using oligo-dT primers and SuperScriptII reverse transcriptase (Invitrogen) per manufacturer's instructions. cDNA from the synthesis reaction was subjected to quantitative, real-time PCR with SYBR Green reporter (Bio-Rad) in the Mx3000P thermal cycler (Stratagene, La Jolla, CA). Cycle threshold values were obtained by normalization to ROX (Stratagene) reference dye. Transcripts were quantified by the Δ-ΔCt method, normalizing to elongation factor EEF1G. Following the reaction, product dissociation curves were analyzed to ensure accurate readings. Primer sequences were: Tau forward (F): GTGGAGGCAGTGTGCAAATA; Tau reverse (R): GCCAATCTTCGACTGGACTC; p38MAPK F: TGAACTTCGCAAATGTATTTATTGGT; p38MAPK R: ATCTGAGTCCAAAACGAGCATCT; EEF1G F: GGCCAAACCAACCGCACC; EEF1G R: CGATGTCACTGTCAGCAAAG. N = 6 for all groups of mice.

### Data Analysis

Data are presented as mean +/- SEM. Statistical analyses between 2 groups were performed by using a two-tailed student's *t* test, whereas comparisons involving multiple groups were performed with a one-way ANOVA followed by Tukey's or Dunnett's post test for multiple comparisons. *P* values ≤0.05 were considered statistically significant. GraphPad Prism Software (SanDiego, CA) was utilized for the analysis.

## Results

First, we utilized MEMRI to determine axonal transport rates in wildtype control (WT) and WT mice at 1 week post-STZ (WT-STZ). The WT-STZ treated mice exhibited significant decreases in axonal transport rates as compared to the WT, Figure 1A. In order to confirm that the deficits in the axonal transport rates were due to STZ-induced hyperglycemia and not STZ-induced toxicity, we treated the mice with insulin pellets. Following 10 days of insulin treatment the mice had an average non-fasting glucose level of <200 mg/dl. The STZ plus insulin treated mice displayed significant recovery in the axonal transport rates, Figure 1B. In addition, we measured axonal transport rates in a mouse model of chronic hyperglycemia. The results indicated a significant reduction in axonal transport rates in mouse model of chronic hyperglycemia (fasting glucose levels 200–550 mg/dl) as compared to the healthy controls (fasting glucose levels <200 mg/dl) Figure 1C. Taken together, the data demonstrated that the deficits in axonal transport rates were due to hyperglycemia in STZ treated mice.

**Figure 2:**
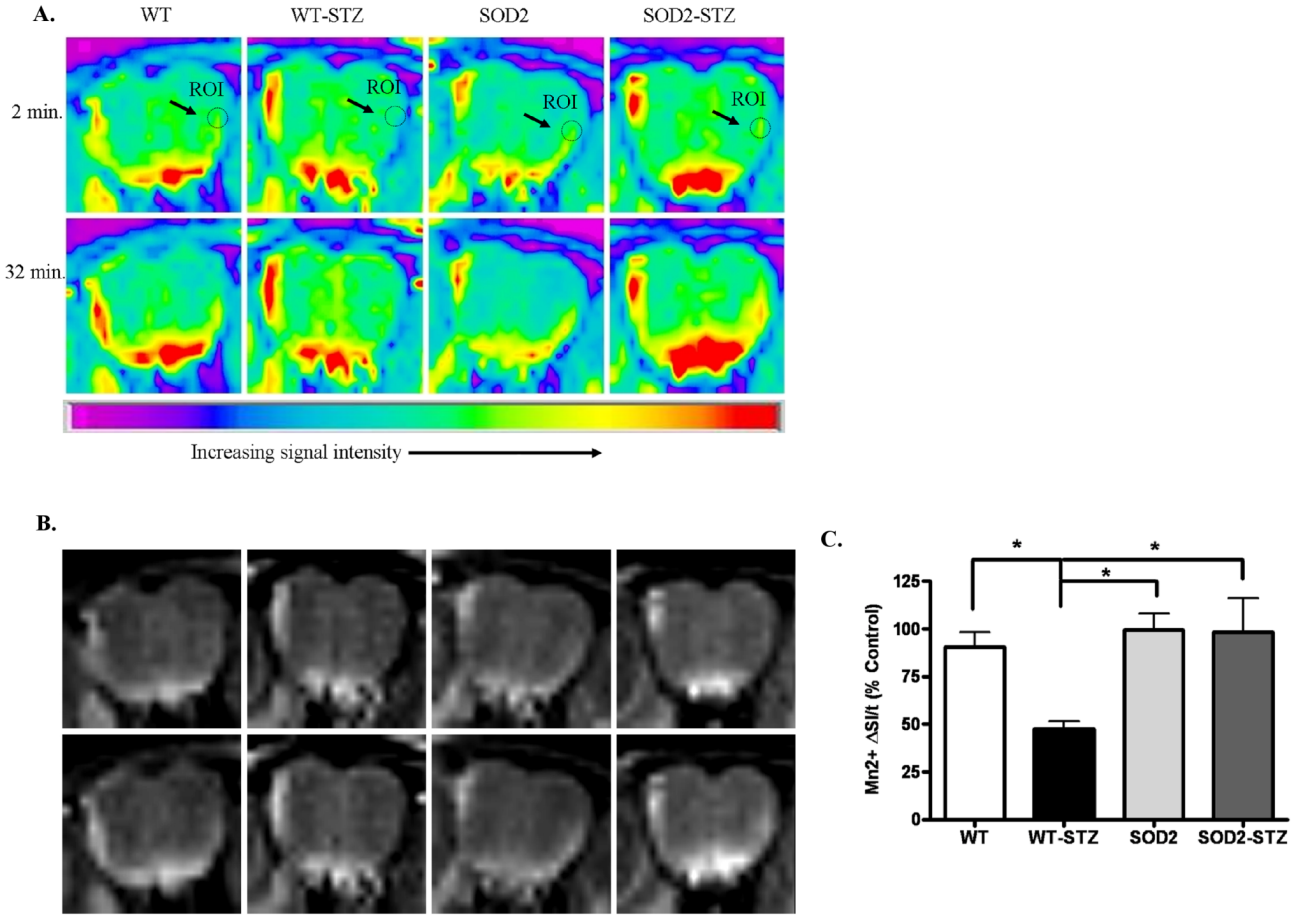
MEMRI experiments demonstrate that the axonal transport deficits in WT-STZ mice recover in SOD2-STZ mice. (A) Pseudo-color MRI images depicting changes in Mn^2+^ signal intensities (yellow color) at the beginning (2minutes) and at the end of the imaging session (32 minutes) at a region of interest (ROI) identified as a circle on the outer olfactory neuronal layer (ONL). Note that the WT, SOD and SOD2-STZ mice exhibit a change from green (2 minute time point) to yellow (32 minute time point) whereas the WT-STZ animals exhibit a light green color at both time points indicating that Mn^2+^ has not traveled to these areas at the same rate. (B) Gray-Scale Image of the same data set in (A). (C) The graph depicts normalized axonal transport rates (% control) in the WT and SOD2 mice treated with vehicle or STZ for a week before *in vivo* axonal transport studies. Twelve mice were used in the WT group and four mice were used in each of WT-STZ, SOD2, and SOD2-STZ groups. Statistical analysis: One way ANOVA, Dunnett's post-test. * p<0.05. SOD2  =  SOD-2 overexpressing mice; SOD2-STZ  =  SOD-2 overexpressing mice treated with STZ.

**Figure 3:**
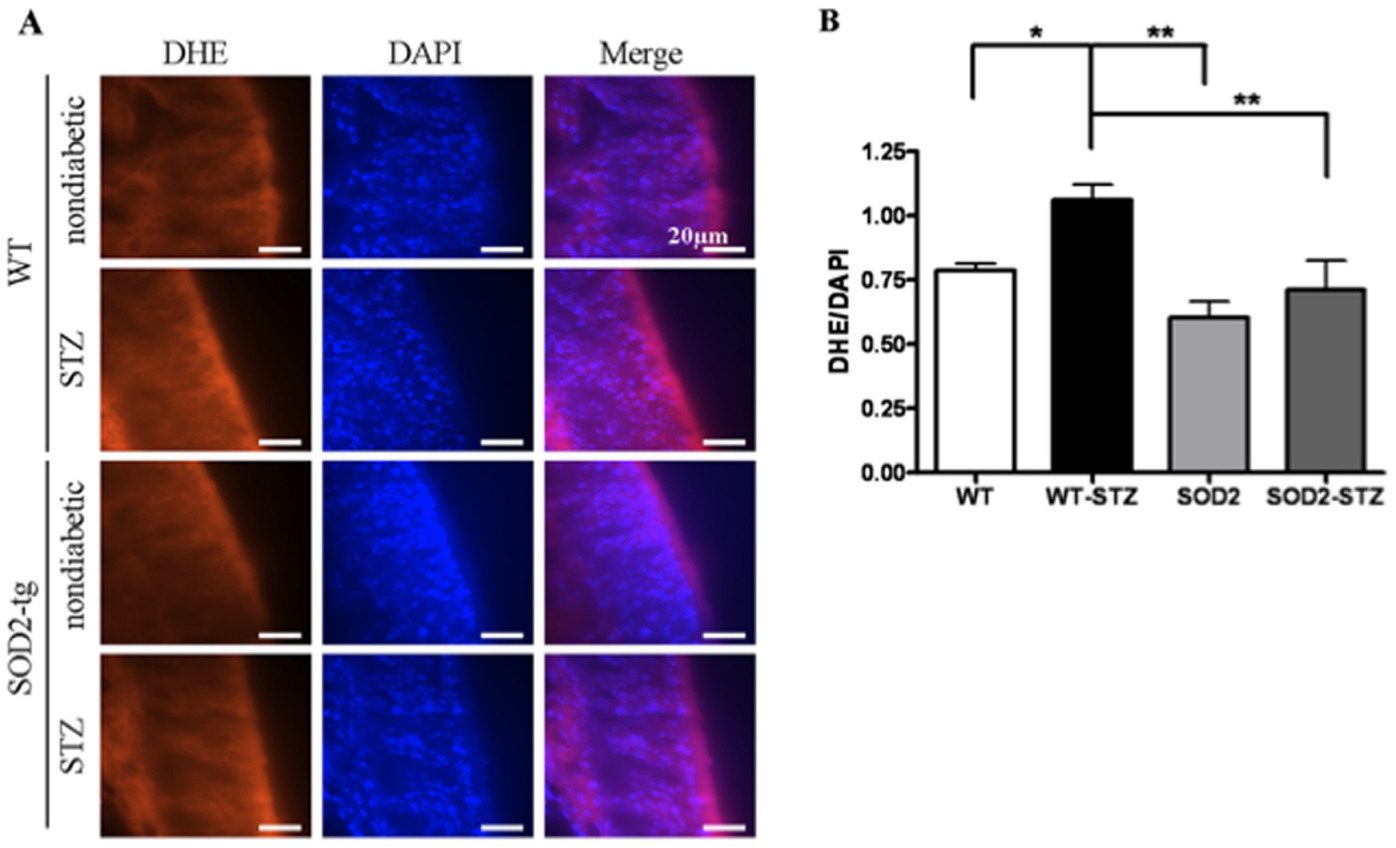
STZ- WT mice depict significantly increased ROS levels that decrease in]SOD2-STZ mice. (A) The images depict nasal cavity sections showing genotypic differences in DHE fluorescence. (B) The graph depicts ratio of DHE and corresponding DAPI fluorescence, which was measured with ImageJ software. Significance was assessed by one way ANOVA with Dunnett's post-test. For WT, WT-STZ, SOD2, and SOD2-STZ n = 4, 6, 4, 4 respectively. ** p<0.01, * p<0.05 Bar = 20 µm.

To determine if axonal transport deficits were associated with hyperglycemia-induced oxidative stress, we measured axonal transport rates with MEMRI in STZ-treated SOD2 overexpressing mice (SOD2-STZ). At 1 week post-STZ treatment, the measured glucose levels of SOD2-STZ, WT-STZ, WT, and SOD2 mice ranged from 283–598 mg/dl, 256–600 mg/dl, 143–177 mg/dl, and 172–189 mg/dl. Figure 2A depicts pseudo colored images (for ease of visualization and qualitative interpretation) and Figure 2B depicts the corresponding grayscale image of the olfactory bulbs of mice. The ROI at the outer layer of the olfactory bulb is indicated by a circle and arrow. At the beginning of the imaging session we observed little Mn^2+^ signal intensity at the ROI (depicted as light green in the ROI, Figure 2A, top row, 2 minute time point). By the end of the imaging session, at 32 minutes, the MRI signal intensity increased for vehicle-treated WT and SOD2 mice as well as for the SOD2-STZ mice (depicted by an increase in yellow color, Figure 2A, bottom row, 32 minute time point) but remained relatively unchanged for the WT-STZ mice as depicted by the light green color at the 32 minute time point. Upon quantification of the Mn^2+^ signal intensity we observed that despite STZ-induced hyperglycemia, axonal transport rates recovered significantly in SOD2 overexpressing mice as opposed to the WT-STZ mice and were indistinguishable from WT and SOD2 controls, Figure 2 C. To determine the association of oxidative stress and axonal transport rates, we utilized dihydroethidium (DHE) staining to measure the levels of ROS in WT, SOD2, SOD2-STZ, and WT-STZ mice. Figure 3A depicts 20 µm thick nasal cavity tissue sections. The first column represents DHE fluorescent images which reflect ROS levels. The second column depicts the corresponding DAPI images and the third column represents an overlay of DHE and DAPI images. The DHE fluorescent section from WT-STZ mice depicted remarkably bright red fluorescence that signified probably high levels of ROS as compared to the WT, SOD2, and SOD2-STZ mice. Image analysis at the ROIs demonstrated that ROS levels are significantly increased in hyperglycemic mice as compared to the controls and that these levels normalized back to control levels in STZ-treated SOD2 overexpressing mice, Figure 3B.

**Figure 4:**
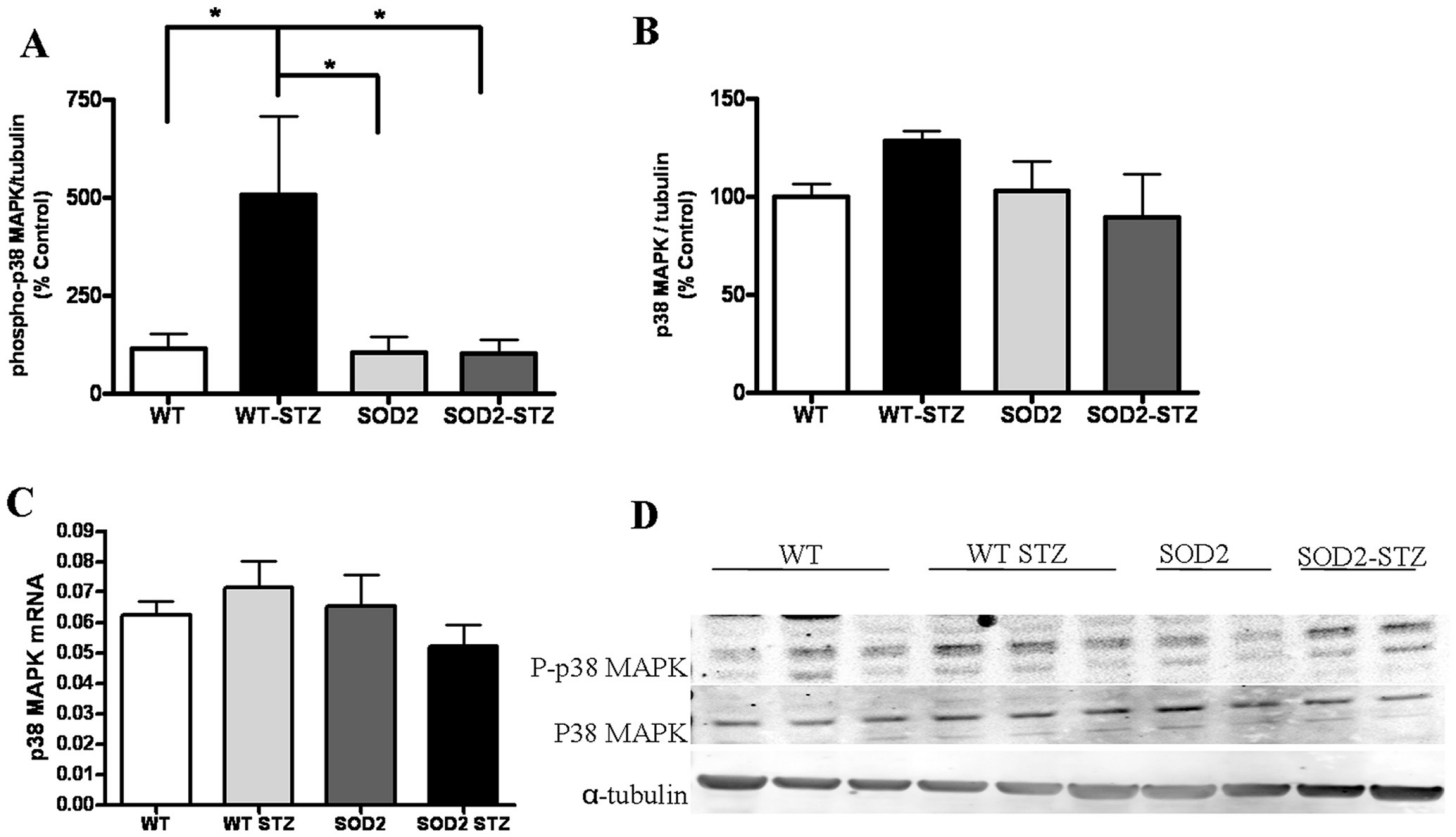
Phosphorylated p38 MAPK significantly increase in WT-STZ mice and recovers in SOD2-STZ mice. Western blotting analysis of OB tissues from WT, WT-STZ, SOD2, SOD2-STZ mice show: (A) changes in phosphorylated p38 MAPK levels (for WT n = 7 and for WT-STZ, SOD2, and SOD2-STZ n = 5), (B) changes in total p38 MAPK protein levels, (for WT and WT-STZ n = 7, for SOD2 n = 6, and for SOD2-STZ n = 5) (C) QPCR experiment using brain homogenates depict relative changes in mRNA levels of p38 MAPK, six mice in each group and (D) representative western blot from OBs homogenates from 1 week vehicle or STZ treated 2-4-month-old mice. The results are normalized to α-tubulin. Statistical analysis: One way ANOVA followed by Dunnett's post test. ** p<0.01, *p<0.05.

We next determined if the p38 MAPK pathway was affected in STZ treated mice in association with the oxidative stress. Western blotting analysis demonstrates significant increases in the levels of phosphorylated p38 MAPK in WT-STZ mice as compared to the WT, SOD2 and SOD2-STZ mice, Figure 4A. The levels of total p38 MAPK protein, however, did not significantly differ in the four groups of mice, Figure 4B. In addition, the p38 MAPK mRNA levels also remained unchanged in all groups of mice, Figure 4C. The phosphorylated and total p38 MAPK protein levels from each sample were normalized to their respective α-tubulin protein amounts; the western blots are shown in Figure 4D. Taken together, the data indicate that phosphorylated p38 MAPK levels increase in association with ROS in hyperglycemic mice with increased oxidative stress and return to wildtype levels in SOD2-STZ mice that have reduced superoxide load despite hyperglycemia.

**Figure 5:**
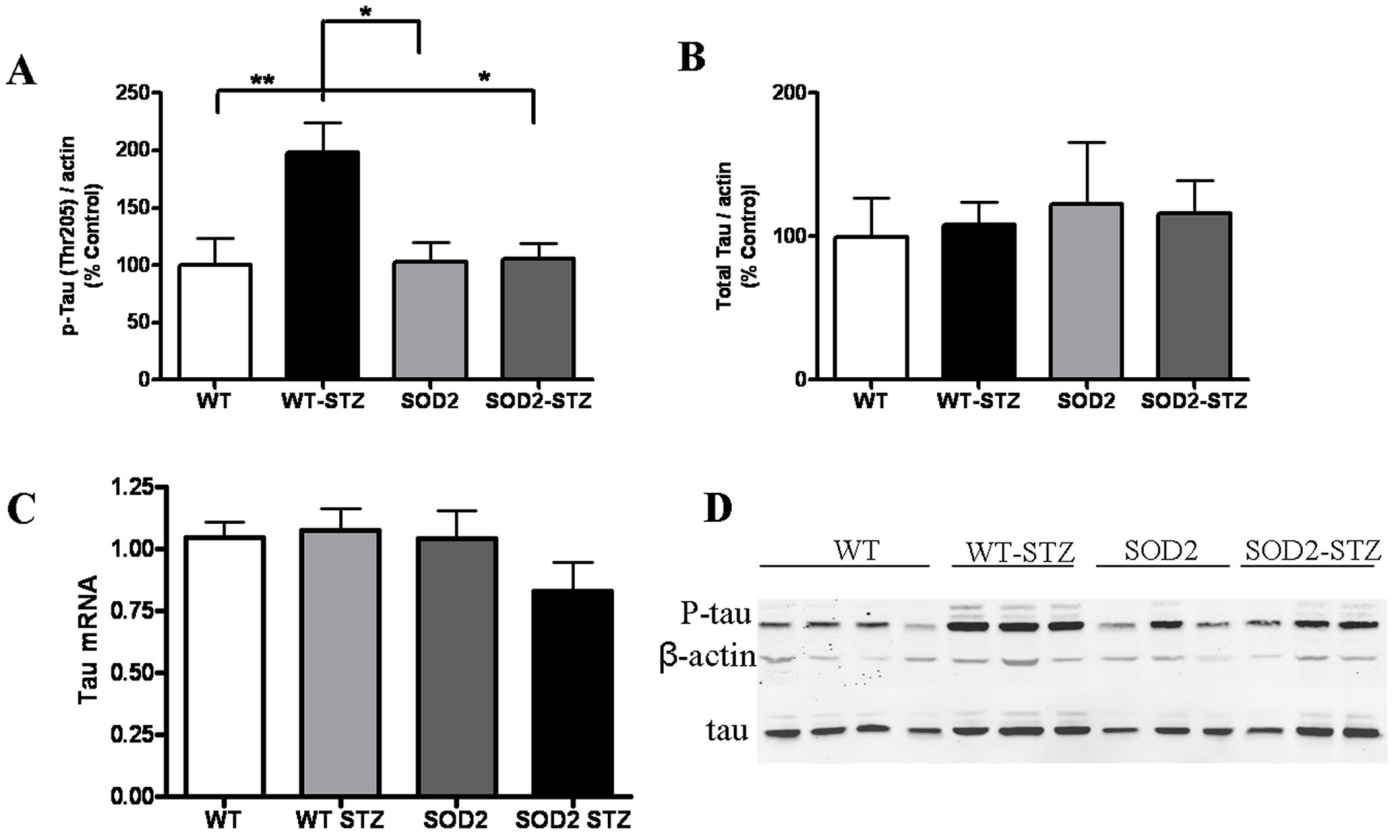
Phosphorylated Tau significantly increase in WT-STZ mice and recovers in SOD2-STZ mice, despite hyperglycemia. Western blotting analysis of OB tissues from WT, WT-STZ, SOD2, SOD2-STZ mice show: (A) changes in site-specific phosphorylation of tau (p-tau) at threonine 205, (seven mice in WT, WT-STZ groups and six in SOD2 and SOD2-STZ groups) (B) changes in total levels of tau protein, (seven mice in WT, WT-STZ groups and six in SOD2 and SOD2-STZ groups) (C) QPCR of brain homogenates depict relative changes in mRNA levels of tau, (six mice used in each of the four groups) and (D) representative western blot from OB homogenates from 1 week vehicle or STZ treated 2-4-month-old mice. The results are normalized to β-actin. Statistical analysis: One way ANOVA followed by Dunnett's post test. ** p<0.01, *p<0.05.

We also assessed the phosphorylation levels of tau at the threonine-205 site. We observed that in WT-STZ mice, there was an increase in the phosphorylation which returned to baseline levels in SOD-STZ animals, Figure 5A. The total tau protein levels and mRNA levels remained unchanged, Figure 5B and Figure 5C respectively. The western blots results were normalized to β-actin with the blots represented in Figure 5D. Together, the data indicates that significantly higher levels of site-specific phosphorylation of tau in conjunction with activation of p38 MAPK occur in hyperglycemic mice (WT-STZ) that have increased superoxide load as compared to the WT, SOD2, and SOD2-STZ mice that have reduced mitochondrial oxidative stress.

## Discussion

The effects of hyperglycemia on the CNS remain poorly understood. Our results indicate that MEMRI noninvasively depicts significant reduction of axonal transport rates in WT-STZ mice during hyperglycemia. Interestingly, the axonal transport rates did not appear to be a function of the levels of hyperglycemia as mice within the range of 200–300 mg/dl or >400 mg/dl exhibited similar decreases in axonal transport as compared to controls (Figure 1A). This abrupt rather than gradual decline could potentially be explained by the fact that although ROS are constantly produced in normal physiology, once a certain threshold of ROS is reached, they become detrimental.

We also determined that the axonal transport rate deficits are associated with activation of p38 MAPK signaling cascade and tau phosphorylation in hyperglycemic mice at 1-week. One possible explanation of these data include a perturbation in calcium (Ca^2+^) homeostasis as Mn^2+^, which is used as a molecular MRI contrast agent, is a Ca^2+^ analogue [16]. Wseveral reports indicated that calcium homeostasis is disturbed with increased levels of intracellular calcium in several cells during long-term diabetes [25]–[26] and increase in T-type calcium currents in peripheral neurons following two weeks of hyperglycemia [27], 5-12 days of hyperglycemia does not cause remarkable alterations in calcium homeostasis [25]. In this work, we performed the MEMRI studies at 1 week post STZ-treatment. which negates the effect of hyperglycemia on possible differential uptake of Mn^2+^ ions by neurons due to altered calcium homeostasis and that our measurements are reflective of the axonal transport of Mn^2+^ as we and others have performed and validated in the past [6]–[7], [18]–[19].

Upon SOD2 overexpression, the deficits in axonal transport recovered in association with restoration of levels of phosphorylated p38 MAPK and tau protein. Because SOD2 is exclusively localized to the mitochondria of SOD2 transgenic mice [28], our data is indicative of the critical role that mitochondrial oxidative stress plays during hyperglycemia as mitochondrial oxidative stress has been implicated in diabetic associated pathologies. For example, SOD2 overexpression in primary dorsal root ganglion cultures or in mice protected cellular injury and prevented development of signs of diabetic neuropathy (DN) respectively [10]. Recently, Munusamy and Mac-Millan-Crow [29] reported that overexpression of SOD2 prevented early stage hyperglycemia-induced mitochondrial injury in normal renal rat proximal tubular cells. Furthermore, overexpression of SOD2 significantly prevented the development of retinopathy in mice by preventing retina from diabetes-induced oxidative damage [30]. Similarly, SOD2 overexpression in heart mitochondria improved mitochondrial respiration, protected heart morphology, and restored cardiac contractility of a diabetic heart [31]. Collectively, the data suggest that hyperglycemia-induced mitochondrial oxidative stress is a major trigger of diabetes-related neuronal injury that can be ameliorated by increasing SOD2 activity.

Our data also depicts hyperglycemia-induced oxidative stress induces activation of p38 MAPK within 1-week of STZ treatment of mice. Previous reports also demonstrate that hyperglycemia-induced oxidative stress causes p38MAPK activation in peripheral neuronal cultures after 16 h of exposure to high glucose, after STZ-treated diabetic rats with 12 weeks of high glucose, and in the sural nerves of type 1 and 2 diabetic patients [11], [12]. We observe that the increased phosphorylation of p38 MAPK in WT-STZ mice is ameliorated in SOD2-overexpressing mice despite the presence of hyperglycemia. Our WB data did not exhibit any alteration in the level of p38 MAPK protein itself. The p38 MAPK WB results were also consistent with Q-PCR data which indicated similar levels of mRNA of p38 MAPK in WT-STZ, vehicle-treated controls, and SOD2-STZ mice. The changes in the phosphorylated levels of p38 MAPK can be ascribed to the alteration of activation status of the kinase at 1-week of hyperglycemia. Similarly, we observed increased phosphorylation of tau at threonine-205 with no changes in tau protein or mRNA levels. Our findings are in agreement with previous reports, [32] where 3-day STZ-treatment causes activation of p38 MAPK in conjunction with tau phosphorylation at threonine-205 in the brains of mice. Additionally, Planel and coworkers, [33] reported significant increase in tau phosphorylation at the threonine-205 site as early as 10 days post-STZ injection in mice brains. Phosphorylation of tau protein at the threonine-205 and serine-202 sites are early markers of tau dysfunction. In our study, however, we did not observe a significant alteration of phosphorylated levels of tau at serine-202 at the 1-week time point. (data not shown). Because tau phosphorylation is believed to destabilize microtubules and hinder fast axonal transport [34], our findings indicate that the mechanism of axonal transport deficits observed involves activation of p38 MAPK pathway in association with phosphorylated tau protein due to acute hyperglycemia-induced oxidative stress.

Current understanding regarding the short-term effects of hyperglycemia is limited. Whereas, renal arterial and carotid blood flow significantly drop and phosphorylated p38 MAPK and ERK1/2 levels significantly increase in aorta and the cortex of the kidney respectively by two weeks of STZ-induced diabetes [35], little is known about the effects of acute hyperglycemia on neurons. One can argue that the deficits in the axonal transport observed in CNS neurons as early as 1 week could be due to STZ-related toxicity. We ruled out the possibility of STZ-induced toxicity by administering insulin for validation experiment (as depicted in Figure 1) following STZ-treatment and observed recovery in axonal transport rates. Thus, the deficits in axonal transport rates that we observed are due to STZ-related insulin deficiency and not toxicity. Additionally, our findings remained consistent in a mouse model of glucose injection-induced hyperglycemia that further confirmed that STZ-induced hyperglycemia was causing axonal transport deficits. However, it should be also noted that it is not clear if similar beneficial effects of the antioxidant SOD2 would be observed in human patients especially because the animal model we used is a transgenic animal that overproduces the antioxidant SOD2 since birth. Clinical trials involving antioxidant therapies to treat long-term diabetes-related complications have resulted in controversial outcomes. Similar controversial effects of antioxidant therapies have been observed in other diseases as well (for e.g., Alzheimer's disease). In such cases, it has been proposed that factors such as the timing of administration or the dosage may have affected the outcome of the trials. Accordingly, if administered early enough (as early as 1 week hyperglycemia) we may be able to observe the beneficial effects and prevention of nerve damage. Our results describe restoration of axonal transport rates following SOD2 overexpression in animal models, and although the model we use does not directly correlate with human disease, similar outcome may be possible in diabetic patients, provided optimization of timing, dosage and potency of antioxidant therapy used.

In conclusion, our results indicate that short-term hyperglycemia-induced oxidative stress induces axonal transport deficits in olfactory receptor neurons in conjunction with activation of p38MAPK pathway and tau phosphorylation. Upon overexpression of SOD2, mitochondrial antioxidant enzyme, hyperglycemia-induced oxidative stress-related axonal transport deficits recover. Research efforts towards developing efficient tools to deliver antioxidants or antioxidant precursors to the cells vulnerable to hyperglycemia may prove valuable in combating hyperglycemia-related complications.
